# Cass County (Indiana) Medical Society

**Published:** 1873-10

**Authors:** J. H. Goodell

**Affiliations:** Sec’y.


### *Cass County (Indiana) Medical Society.*

A number of the physicians of Cass county, Indiana, met in the Court House, in Logansport, and organized a County Medical Society, by adopting a constitution and electing the following members officers for the ensuing year:

*President*—J. A. Adrain, Onward.

*Vice-President*—W. H. Bell, Logansport.

*Secretary*—J. H. Goodell, Walton.

*Treasurer*—J. M. Justice, Logansport.

*Censors*—Asa Coleman, Logansport; James Thomas, Royal Center; I. B. Washburn, Logansport.

The Society adjourned to meet in November.

J. H. GOODELL, *Sec'y.*

